# Epidemiology of Non-Hodgkin’s Lymphoma

**DOI:** 10.3390/medsci9010005

**Published:** 2021-01-30

**Authors:** Krishna C. Thandra, Adam Barsouk, Kalyan Saginala, Sandeep Anand Padala, Alexander Barsouk, Prashanth Rawla

**Affiliations:** 1 Department of Pulmonary and Critical Care Medicine, Sentara Virginia Beach General Hospital, Virginia Beach, VA 23455, USA; 2 Sidney Kimmel Cancer Center, Jefferson University, Philadelphia, PA 19107, USA; adambarsouk@comcast.net; 3 Plains Regional Medical Group Internal Medicine, Clovis, NM 88101, USA; drsaginala@gmail.com; 4 Department of Medicine, Nephrology, Medical College of Georgia, Augusta University, Augusta, GA 30912, USA; spadala@augusta.edu; 5 Hematologist-Oncologist, Allegheny Health Network, Pittsburgh, PA 15212, USA; alexbarsouk@comcast.net; 6 Department of Medicine, Sovah Health, Martinsville, VA 24112, USA; rawlap@gmail.com

**Keywords:** NHL, non-Hodgkin’s lymphoma, epidemiology, incidence, prevalence, mortality, prevention, risk factors

## Abstract

Non-Hodgins’s lymphoma (NHL) is the most common hematological malignancy worldwide, accounting for nearly 3% of cancer diagnoses and deaths. NHL is the seventh most prevalent cancer and has the sixth highest mortality among cancers in the US. NHL accounts for 4% of US cancer diagnoses, and incidence has increased 168% since 1975 (while survival has improved 158%). NHL is more common among men, those >65 years old, and those with autoimmune disease or a family history of hematological malignancies. NHL is a heterogenous disease, with each subtype associated with different risk factors. Marginal zone lymphoma (MZL) is strongly associated with Sjogren’s syndrome (SS) and Hashimoto’s thyroiditis, while peripheral T-cell lymphoma (PTCL) is most associated with celiac disease. Occupational exposures among farm workers or painters increases the risk of most of the common subtypes. Prior radiation treatment, obesity, and smoking are most highly associated with diffuse large B-cell lymphoma (DLBCL), while breast implants have been rarely associated with anaplastic large cell lymphoma (ALCL). Infection with Epstein–Barr Virus (EBV) is strongly associated with endemic Burkitts lymphoma. HIV and human herpes virus 8 (HHV-8), is predisposed to several subtypes of DLBCL, and human T-cell lymphoma virus (HTLV-1) is a causative agent of T-cell lymphomas. Obesity and vitamin D deficiency worsen NHL survival. Atopic diseases and alcohol consumption seem to be protective against NHL.

## 1. Introduction

Non-Hodgins’s lymphoma (NHL), the most common hematological malignancy worldwide, refers to a diverse class of B-cell and T-cell proliferations. NHL is differentiated from Hodgkin’s lymphoma by different clinical characteristics and the absence of Reed–Sternberg cells and Cd15 and Cd30 staining on histology. While there are over 40 major subtypes, the most common types include indolent follicular lymphoma (FL) and aggressive diffuse large B-cell lymphoma (DLBCL). Each type is associated with unique driver genetic mutations (e.g., 14:18 translocation in FL, 11:14 translocation in Mantle Cell, 8:14 in Burkitts lymphoma ) and unique risk factors (Epstein–Barr Virus (EBV) for Burkitt’s lymphoma, human T-cell lymphoma virus (HTLV-1) for T-cell lymphoma) [1]. Population-based cancer registries, such as those compiled in the 2018 GLOBOCAN, do not distinguish NHL subtypes based on the extensive WHO classifications, making global epidemiological data on NHL subtypes limited [2]. Due to the heterogeneity of NHL, the International Lymphoma Epidemiology Consortium (InterLymph) was established in 2001 to elucidate subtype-specific risk factors and disease mechanisms [3]. Global epidemiology statistics as well as common risk factors identified by InterLymph, based on meta-analysis data, are summarized below.

## 2. Epidemiology

### 2.1. Incidence

**Figure 1:**
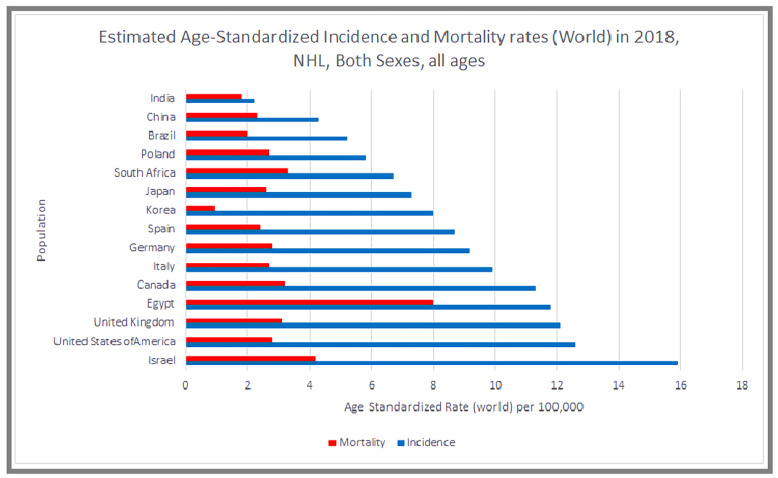
Bar chart showing estimated age-standardized incidence and mortality rates (World) in 2018, non-Hodgkin’s lymphoma, all sexes, all ages. Data obtained from Globocan 2018.

According to the latest GLOBOCAN data, an estimated 509,600 new cases of NHL were diagnosed globally in 2018, comprising 2.8% of worldwide cancer diagnoses. The global age standardized risk of NHL was 6.7 among men and 4.7 among women, translating to a 0.72% and 0.35% cumulative lifetime risk for men and women, respectively. The incidence in high and low/medium human development index nations, respectively, was 7.8/100,000 and 4.3/100,000 among men and 5.6/100,000 and 2.9/100,000 among women (Figure 1) [4].

According to the latest World Health Organization (WHO) classification, the most common NHL in Western countries is DLBCL, accounting for around 31% of adult cases. Other common aggressive B-cell subtypes include Mantle Cell Lymphoma (MCL) (6% of cases) and BL (2% of cases). Among indolent B-cell NHL, FL accounts for 22% of cases in the Western world, followed by marginal zone lymphoma (MZL) (8% of cases), chronic lymphocytic leukemia/small-cell lymphocytic lymphoma (CLL/SLL) (6% of cases) and lymphoplasmacytic lymphoma (LPL) (1% of cases). Common T-cell lymphomas make up only 10–15% of NHL diagnoses and include peripheral T-cell lymphoma (PTCL) (6% of cases) and cutaneous T-cell lymphoma (CTCL) (4% of cases) [1].

**Figure 2:**
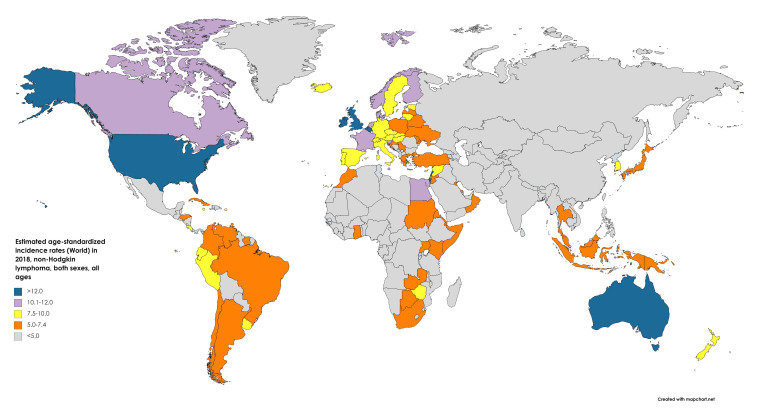
Map showing estimated age-standardized incidence rates (ASR) for non-Hodgkin’s lymphoma worldwide in 2018, all sexes, including all ages. Created with mapchart.net. Data obtained from Globocan 2018.

An estimated 77,200 new cases of NHL were diagnosed in 2020 in the US, accounting for 4.3% of cancer diagnoses (the seventh most common cancer diagnosis). The most recent reported incidence was 18.6/100,000 in 2017, a 168% increase from the earliest reported incidence of 11.1/100,000 in 1975 [5]. Figure 2 shows age-standardized incidence rates for NHL worldwide in 2018.

### 2.2. Mortality

**Figure 3:**
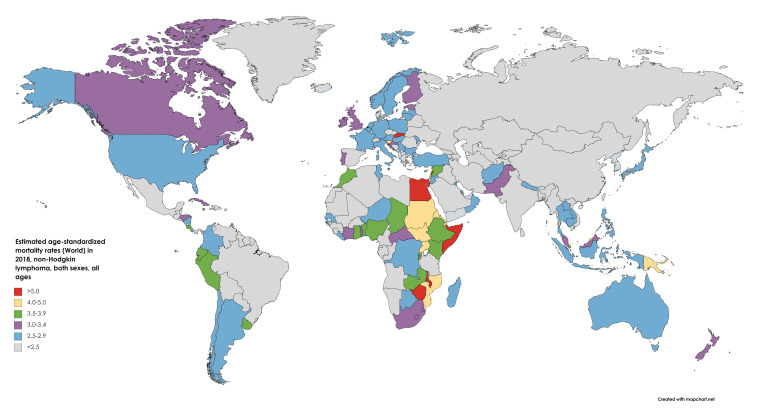
Map showing estimated age-standardized mortality rates (ASR) for non-Hodgkin’s lymphoma worldwide in 2018, all sexes, including all ages. Created with mapchart.net. Data obtained from Globocan 2018.

An estimated 248,700 global deaths were attributable to NHL in 2018 (See Figure 3), accounting for 2.6% of all oncological mortality. Men had a 0.33% cumulative lifetime risk of NHL mortality, while the risk for women was estimated at 0.21%. The mortality in both high and low/medium HDI nations was 3.2/100,000 among men, and 2.0/100,000 and 1.9/100,000 among women, respectively [4].

The mortality in the US was an estimated 19,900 in 2020, accounting for 3.3% of all cancer deaths (making NHL the sixth leading cause of oncological mortality). The most recent reported mortality was 5.1/100,000 in 2018, down from a peak of 8.9/100,000 in 1997. The death rate was for men was 7.0/100,000, significantly greater than the 4.1 reported in women [5].

### 2.3. Survival

The NHL 5-year survival from 2010 to 2016 in the US was 72.7%. This is a 158% improvement from the earliest reported 5-year survival in 1975: 46%. The 5-year survival for stage I disease at diagnosis is 83.5% (25% of all diagnoses), while the survival for stage IV disease is 63.3% (33% of diagnoses). The discovery and implementation of improved stem cell transplant techniques, new cytotoxic regimens, anti-Cd-20 antibodies such as rituximab, and most recently, targeted therapies like bcl-2 inhibitor venetoclax and PD-1 inhibitor pembrolizumab have significantly improved outcomes across stages for relapsed/refractory disease [5].

## 3. Non-Modifiable Risk Factors

Table 1 indicates modifiable and non-modifiable risk factors.

### 3.1. Age

The average age of diagnosis was 67, with 57% of diagnoses made in those >65 years old. The mean age of death was 76 years old, with 78.5% of deaths occurring in those >65 years old. Several subtypes of NHL have a younger age of diagnosis. BL has a bimodal age distribution, with the endemic form, associated with EBV, most common in children and young adults. DLBCL, LPL and anaplastic large cell lymphoma can also occur in children. NHL associated with underlying inflammatory processes (such as MZL) or immunosuppression (DLBCL) often occur in adults younger than 65.

### 3.2. Gender

Globally, men have over double the cumulative lifetime risk of developing NHL. In high HDI nations, men are 39% more likely to be diagnosed and 60% more likely to die of the disease. Greater prevalence of certain risk factors, such as obesity, HIV, and chemical exposure among men may help explain the increased risk.

### 3.3. Race/Ethnicity

In the US, white and non-Hispanic people are at highest risk of NHL, while Asian/Pacific Islander, American Indian and black populations are at the lowest risk. The incidence out of 100,000 was 25.0 and 17.0 in white men and women, respectively, compared to 10.8 and 10.9 among American Indians, 16.5 and 11.1 in Asian/Pacific Islanders, 17.7 and 12.5 in African Americans, and 20.9 and 15.9 in Hispanics [5]. However, the T-cell lymphoma mycosis fungoides shows higher incidence among African Americans [3]. East Asian nations, in particular Japan, as well as nations in West Africa and the Caribbean, have higher rates of human T-cell lymphoma virus (HTLV-1), and subsequently higher incidence of T cell lymphoma. Similarly, children in subequatorial Africa are at higher risk of endemic Burkitt Lymphoma from Epstein–Barr Virus (EBV), with an interplay with malaria infection suspected. The discrepancies in viral NHL risk across ethnicities and geography remains unelucidated [6].

### 3.4. Family History

Family history of hematological malignancy has been implicated as a risk factor across subtypes of NHL. Across subtype-specific meta-analyses undertaken by InterLymph, first-degree family history was associated with an OR of 1.95 in DLBCL [7], an OR of 1.64 in LPL [8], an OR of 1.99 in FL [9], an OR of 2.16 in CLL/SLL [10], an OR of 1.99 in MCL [11], OR of 1.90 in MZL [12], and an OR of 1.92 in PTCL [13].

### 3.5. Autoimmune Diseases

Autoimmune diseases such as Sjogren’s syndrome (SS), systemic lupus erythematosus (SLE), celiac disease and scleroderma have been associated with various subtypes of NHL. The strongest risk factor for MZL, in particular, is chronic inflammation, either due to autoimmune attack or infection. As such, the OR for developing MZL in those with SS (parotid MZL) and SLE are 38.1 and 6.54, respectively. MZL of the thyroid (thyroid mucosa associated lymphoid tissue or MALToma) is also associated with Hashimoto’s thyroiditis. The OR for MZL with other B-cell activating autoimmune diseases is 5.46 [12]. DLBCL is associated with all of the aforementioned autoimmune diseases to a lesser degree, with OR ranging from 2.09 for celiac disease to 8.77 for SS [7]. PTCL is most strongly associated with celiac disease (OR = 14.8) due to Cd4 T cell involvement, as well as SLE (OR = 3.90) and other T-cell activating disease (OR = 1.95) [13]. Scleroderma was most highly associated with BL (OR = 20.2) [14]. Atopic diseases such as allergies, hay fever and eczema were found to decrease the risk of most common subtypes of NHL [15].

### 3.6. Immunosuppression

Certain subtypes of NHL have been associated with immunosuppressive conditions. The immunodeficiency associated subtype of BL has been highly associated with human immunodeficiency virus (HIV) and solid organ transplant [14]. Among those with organ transplants in the US, the risk of BL is 23× higher, in part due to increased risk of infection with EBV. The risk is likewise heightened for those undergoing immunosuppressive therapies for autoimmune diseases [16].

## 4. Modifiable Risk Factors

### 4.1. Radiation

Risk of NHL seems to be increased following radiation for solid malignancies. A US study reported a relative risk (RR) of 1.13 among 1.5 million one-year cancer survivors who received radiotherapy. Radiation for non-small cell lung cancer had the highest RR of 1.53, likely due to proximity to the mediastinum (a common location for DLBCL) [17]. Chemotherapy regimens for cancers have also been suggested to increase the risk of NHL later in life, though large-scale population studies quantifying the risk could not be found, perhaps due to constant evolution and heterogeneity of cytotoxic agents used [18].

### 4.2. Chemical Exposure

Exposure to various occupational chemicals and tobacco has been linked with several subtypes of NHL. Working on a farm or as a painter is associated with an increased risk of NHL (OR = 1.28, 1.22, respectively) [15]. For DLBCL in particular, working on a farm (OR = 1.78), as a seamstress and embroider (OR = 1.49), hairdresser (OR = 1.65), or equipment operator/driver (OR = 1.58), in particular mediastinal DLBCL [7]. Working as a spray painter was associated with FL with an OR of 2.66 [9]. Certain subtypes of PTCL are associated with a >40-year history of smoking (OR = 1.76), working with electrical fitters (OR = 4.08 for anaplastic large cell lymphoma (ALCL) and OR = 5.45 for angioimmunoblastic T-cell lymphoma (AITL)), or working with textiles (OR = 2.60 for ALCL) [13]. Mycosis fungoides, the most common type of cutaneous T-cell lymphoma, was associated with working in farming (OR = 2.37), painting (OR = 3.71), woodworking (OR = 2.20), or carpentering (OR = 4.07) [19,20].

### 4.3. Obesity

Among the InterLymph studies, obesity was not found to be a significant risk factor for most NHL subtypes. However, BMI > 25 kg/m^2^ had an OR of 1.95 with PTCL and 1.32 with DLBCL [13]. In a separate study, a BMI > 30 kg/m^2^ was found to be associated with inferior NHL survival (HR = 1.32) [21].

### 4.4. Tobacco Smoking and Alcohol

Most NHL subtypes do not have a significant association with smoking tobacco [13]. However, cigarette smoking is associated with an increased risk of CNS, testicular and cutaneous DLBCL [5]. Alcohol consumption was associated with decreased risk of most common subtypes of NHL [13]. While the association between cigarette smoking and inferior NHL survival did not reach statistical significance, survival was worsened for former smokers (HR = 1.59) and current smokers (HR = 1.50) as compared to non-smokers. Greater number of pack-years and less time since quitting were associated with worse survival as well [19].

### 4.5. Breast Implants

Anaplastic large cell carcinoma (ALCL) has been, in rare cases, associated with breast implants. Before 2018, 516 pathologically confirmed cases of breast implant associated ALCL were documented around the world, with a mean implant duration of 7–13 years (the mean in the US was reported as 10.7 years). Of all these cases, 16 fatalities were reported in the study [22,23].

### 4.6. Vitamin D Deficiency

Increased sun exposure was shown to decrease the risk of DLBCL, FL, CLL/SLL and ALCL, likely by stimulating production of vitamin D3, a potent immune modulator [24]. However, vitamin D supplementation did not significantly affect NHL risk in a meta-analysis [25]. Higher vitamin D levels were associated with improved lymphoma survival among Norwegian patients [26], and higher vitamin D levels at diagnosis decreased cancer fatality rates (HR = 0.59) after a mean 14.4 year follow-up time [27]. Studies from other nations also found that vitamin D deficiency was associated with inferior survival in CLL/SLL [28], DLBCL, and T-cell lymphoma [29]. A randomized control study at the Mayo clinic investigating vitamin D as a chemotherapy adjunct for NHL is currently recruiting.

## 5. Infections

Table 2 below indicates subtypes of NHL associated with various infections.

### 5.1. Epstein–Barr Virus (EBV)

EBV, also known as HHV-4, is strongly associated with endemic BL, which most often presents as a jaw-lesion (or kidney) on children in developing nations. In studies in Egypt and Brazil, 87% of BL patients were found to be EBV positive. EBV was also found to account for 74% of all childhood malignancies in subequatorial Africa. EBV increases the risk of *MYC* translocation from chromosome 8 to an immunoglobulin locus on chromosome 14, rendering the oncogene constitutively active [30].

### 5.2. Human Immunodeficiency Virus (HIV)

HIV has been implicated in the development of multiple subtypes of NHL, most commonly DLBCL, as well as the rarer but fatal primary brain lymphoma (PBL). A meta-analysis of prospective studies from Europe reported the incidence of NHL was 463/100,000 person-years for those with untreated HIV, and 205/100,000 for those treated (50–100 times above the average risk) [31]. Autologous stem-cell transplant along with multi-agent antiretroviral therapy have been shown to dramatically improve HIV-associated NHL prognosis [32]. With the advent of antiretroviral therapy and prophylaxis, HIV transmission has markedly decreased in the Western world, though it remains a public health crisis in many developing nations.

### 5.3. Human T-Cell Lymphotropic Virus (HTLV-1)

HTLV-1 has been proven to cause T cell lymphomas such as PTCL and Mycosis Fungoides HTLV-1 is also known to cause a debilitating disease called HTLV-1-associated myelopathy/tropical spastic paraparesis (HAM/TSP). Among patients with this disease, the prevalence of T cell lymphoma was 3%. Co-infection of HTLV-1 and HIV has been shown to accelerate development of AIDS and increase risk of T cell lymphoma [13,33,34].

### 5.4. Human Herpes Virus 8 (HHV-8)

Primary effusion lymphoma (PEL) is a variant of DLBCL primarily seen in the immunosuppressed with high HIV viral loads and low CD4+ T-cell counts. PEL is always associated with HHV-8, the herpes virus more commonly implicated in Kaposi Sarcoma in those with AIDS. Of PEL cases, 80% also show concomitant EBV infection. HHV-8-associated large B-cell Lymphoma (HHV-8-LBL) is a variant of PEL that demonstrates both lymphadenopathy and lymphomatous effusions in bodily cavities [35].

### 5.5. Hepatitis C

Hepatitis C (HCV) seropositivity has been associated with increased risk of NHL (OR = 1.81) [15]. Although HCV established chronic infection in 80–90% of patients, unlike the aforementioned viruses, HCV infection has not been implicated as a “driver” of NHL development. Nonetheless, the inflammatory modulation that comes with HCV increases the risk of DLBCL (OR = 2.33) [7], CLL/SLL (2.08) [10], MZL (3.04) [12], and LPL (2.70) [8].

### 5.6. Helicobacter Pylori

Gastric (MALT-gastric mucosa-associated lymphoid tissue) lymphoma is a form of non-Hodgkin’s lymphoma affecting the stomach. There is limited evidence available, but available case-control studies showed association of H. pylori infection and gastric non-Hodgkin’s lymphoma with odds ratio of 6.3%. Eradication of H. pylori with therapy was associated with high rates of remission of the MALT lymphoma [36].

## 6. Conclusions

NHL is a heterogenous group of diseases which constitute the most commonly diagnosed hematological malignancy worldwide, comprising nearly 3% of all cancer diagnoses. In the US, NHL is the seventh most common and sixth most deadly malignancy. NHL is more common among men >65 years old of European and Hispanic descent. While survival has improved in the Western world with new therapeutics, prevention measures aimed at the diverse array of NHL risk factors could significantly reduce the global cancer burden. In particular, reducing chemical exposure in the workplace, tobacco smoking, obesity, and transmission of viruses such as HIV, EBV, HTLV-1 and HCV, and increasing sun exposure and vitamin D supplementation, are strong targets for public health initiatives aimed at NHL prevention.

## Figures and Tables

**Table 1:** Modifiable and non-modifiable risk factors.

Non-Modifiable Risk Factors	Modifiable Risk Factors
Age	Radiation
Gender	Chemical Exposure
Race/Ethnicity	Obesity
Family History	Tobacco smoking and alcohol
Autoimmune Diseases	Breast Implants
Immunosuppression	Vitamin Deficiency

**Table 2:** Subtypes of non-Hodgkin’s lymphoma (NHL) associated with infections.

Infection	Sub Types of NHL
EBV	Burkitt’s lymphoma
HIV	DLBCL, Primary brain lymphoma
HTLV-1	DLBCL
HHV-8	Primary effusion lymphoma
Hepatitis C	DLBCL
Helicobacter Pylori	MALT
